# Bone Remodeling in Mandible of Wistar Rats with Diabetes Mellitus and Osteoporosis

**DOI:** 10.1055/s-0042-1745768

**Published:** 2022-07-04

**Authors:** Nike Hendrijantini, Yonatan Christian Suisan, Rizko Wira Artha Megantara, Bambang Agustono Satmoko Tumali, Mefina Kuntjoro, Muhammad Dimas Aditya Ari, Ratri Maya Sitalaksmi, Guang Hong

**Affiliations:** 1 Department of Prosthodontic, Faculty of Dental Medicine, Universitas Airlangga, Surabaya, Indonesia; 2 Resident of Prosthodontics, Faculty of Dental Medicine, Universitas Airlangga, Surabaya, Indonesia; 3 Division for Globalization Initiative, Graduate School of Dentistry, Tohoku University, Sendai, Japan

**Keywords:** diabetes mellitus, osteoporosis, bone remodeling, medicine

## Abstract

**Objectives** This study aimed to determine some of bone molecular expressions and its possible bone remodeling pathway between diabetes mellitus (DM) and osteoporosis model in the mandibular bone of Wistar rats.

**Materials and Methods** Twenty-seven female Wistar rats were divided randomly into control and treatment groups. Treatment groups were injected with streptozotocin intraperitoneally to induce DM (P1) and underwent bilateral ovariectomy to generate osteoporosis (P2). All groups were terminated after 12 weeks. Immunohistochemical and hematoxylin–eosin staining were performed to determine the expression of Runt-related transcription factor 2 (RUNX2), Osterix, vascular endothelial growth factor (VEGF), receptor activator of nuclear factor κB ligand (RANKL), osteoprotegerin (OPG), tartrate-resistant acid phosphatase (TRAP), and observed the osteoblast and osteoclast. Statistical analysis was performed using one-way analysis of variance.

**Results** The lowest mean of RUNX2 and VEGF expression was found in the P2 group. The lowest mean of Osterix expression was found in the P1 group. Both P1 and P2 groups of osteoblast/osteoclast ratio were decreased. There were no significant differences in the expression of TRAP between all groups; however, increased expression of RANKL/OPG ratio was only found in the P2 group.

**Conclusion** DM and osteoporosis induce changes in the bone remodeling pathway which are represented by a decrease in osteoblast biomarkers and an increase in osteoclast biomarkers.

## Introduction

Implant failure can occur due to low bone mineral density which results in imperfect osteointegration between the implant and the bone. Many factors can cause a decrease in bone mineral density, one of which is systemic diseases, such as diabetes mellitus (DM) and osteoporosis. Based on previous studies, the percentage of implant failure in DM conditions is around 14.3% and osteoporosis is around 10.9%. 1 2 3

DM is a chronic disease that occurs when the pancreas is unable to produce enough insulin or when the body is unable to effectively use the insulin it produces. The prevalence of DM worldwide in 2014 reached 8.5% of the entire adult population, and in Indonesia, the prevalence of DM in 2013 in adult individuals reached 6.9%. This condition that can lead to failure of dental implant treatment for it is associated with prolonged wound healing, the prevalence of microvascular disease (chronic hyperglycemia), and impaired response to infection. Histological analysis showed that bone implant contact in diabetic experimental animals was smaller than nondiabetic. The rates of mineral apposition on new bone formation and bone density around dental implants were significantly lower in uncontrolled diabetic experimental animals. 4 5 6 7 8 9

DM causes changes in bone remodeling and reduced remineralization which results in poor osseointegration, therefore, lead to osteoporosis. 10 11 12 13 14 Osteoporosis is a progressive systemic bone disease characterized by reduced bone mass or density and microarchitecture deterioration of bone tissue. The Indonesian Osteoporosis Association in 2007 reported that the proportion of osteoporosis sufferers in the population over 50 years of age ranges from 32.3% in women and 28.8% in men. The etiology of osteoporosis is an increase in bone resorption and a decrease in bone formation, an imbalance between osteoclast and osteoblast activity. The activity of osteoclasts and osteoblasts is influenced by the balance between osteoprotegerin (OPG), receptor activator of nuclear factor κB ligand (RANKL), and receptor activator of nuclear factor-κB (RANK) which directly plays an important role in the physiological process of bone remodeling. This complex of three molecules functions as a receptor and a ligand. The interaction between RANK–RANKL signifies the initiation of osteoclastogenesis and activation of osteoclasts. The interaction between OPG and RANKL serves to offset the effect of the RANK–RANKL interaction. 5 15 16 17

Bone resorption is related to osteoclast and osteoblast activity. Many biomarkers can be used to see the activity of these cells. A decrease in the osteoblast/osteoclast ratio, an increase in the RANKL/OPG ratio, and the level of tartrate-resistant acid phosphatase (TRAP) secreted by osteoclasts during the bone resorption process are important biomarkers that can indicate the activity of these cells. 18 19

In an *in vivo* study, hyperglycemia, one of the characteristics of DM, was associated with decreased expression of Runt-related transcription factor 2 (RUNX2), where the transcription factor of this gene is the “master regulator” of osteoblast development. 20 Osterix is another transcription factor that plays important role in osteoblast differentiation and matrix mineralization. Osterix promotes osteoblasts in the late maturation stage and inhibits their proliferation by regulating the canonical Wnt/β-catenin pathway. 21

One of the important steps in the success of bone formation is angiogenesis at the wound site. Vascularization provides not only a supply of nutrients and growth factors but also the migration of osteoprogenitors and osteoclasts from hematopoietic precursors. The process of angiogenesis is impaired in a diabetic mouse model. Decreased vascular endothelial growth factor (VEGF) expression is also an indication of impaired angiogenesis. Skeletal vascularization and bone remodeling including differentiation of osteoblasts and bone resorption by osteoclasts are important keys to bone healing. VEGF is known to be a major angiogenic and osteogenic promoter to facilitate bone regeneration. 22

This study will inspect at the differences in osteoblast/osteoclast ratio, RANKL/OPG ratio, TRAP, RUNX2, Osterix, and VEGF expressions in Wistar rats with DM and osteoporosis. In osteoporosis and DM conditions, there is a change in the remodeling process in the bones that leads to a decrease in bone density. This decrease can affect the success of dental treatment, especially dental implants. In these two systemic conditions, we will compare how the bone remodeling process is seen based on markers of osteoblastogenesis and osteoclastogenesis so that we can find out which conditions are most at risk for dental implant failure. The results of this study are expected to help determine the optimal dental implant treatment plan in this condition.

## Materials and Methods

### Materials and Instrumentations

Materials for the preparation of experimental animal models of DM: streptozotocin (STZ), citrate buffer. Materials for termination of experimental animals and taking of research specimens: 10% ketamine, 1 mL xylazine 2%, 4% paraformaldehyde, 10% buffered formalin, ethylenediaminetetraacetic Acid (EDTA). Materials for staining and reading hematoxylin–eosin (HE) are slide, xylol solution, absolute ethanol, 70% ethanol, Meyer's HE solution, eosin solution, adhesive liquid (DPX), cover slip, and fluorescent microscope. Materials for immunohistochemical (IHC) staining are xylol solution, absolute ethanol, 70% ethanol, aquabidest, H _2_ O _2_ 3%, phosphate buffered saline solution, 0.025% trypsin, ultra V block, monoclonal, primary antibody enhancer, and horseradish peroxidase.

The instruments used in the study were a 3-mL syringe, 10-mL syringe, sterile cotton, gauze, dental tweezers, chirurgie tweezers, surgical scissors, a scalpel handle, scalpel blade, sewing needle, absorbable sewing thread, arterial clamp, and Nikon H600L light microscope.

### Animals and Study Design

All animal experiments and procedures were approved by Faculty of Dental Medicine Health Research Ethical Clearance Commission, Universitas Airlangga (553/HRECC.FODM/VIII/2019). Eight- to 10-week-old female Wistar rats ( *n* = 27 [determination of the number of samples in our study using Lameshow. From the calculation, the minimum number of samples per group of 8.9 is fulfilled to 9]; weighing 150–200 g) were acclimatized in Stem Cell Research and Development Centre, Institute of Tropical Disease animal facility under a 12-hour light–dark cycle with ad libitum access to standard chow and water. Wistar rats were randomly divided into three groups: diabetic, osteoporotic, and control groups. Diabetic group (P1; *n* = 9) was injected with STZ intraperitoneally (20 m/kg body dissolved in sodium citrate buffer pH 4.5) for 5 consecutive days. Based on our previous experiments using STZ and alloxan, the lowest animal mortality rate was found with multiple low doses of STZ. To calibrate the hyperglycemic condition in the model, blood glucose checks are performed periodically every day using a glucometer through blood taken from rat tails. Blood glucose was monitored 1 day after the STZ injection using glucometer. Animals were considered diabetic when their blood glucose levels were > 300 mg/dL, they were maintained for 4 weeks. After injection of STZ with multiple low doses for 5 days and waiting for 4 weeks, the general condition of the experimental animals was in good condition. There was no significant change in weight loss. Osteoporotic group (P2; *n* = 9) was underwent bilateral ovariectomy and maintained for 12 weeks to induce osteoporosis and have blood glucose level < 100 mg/dL. Control group ( *n* = 9) was left without any intervention and have blood glucose level < 100 mg/dL This group was maintained for 1 week. All rats were killed and mandibles were obtained for histological staining and IHC evaluation.

### Hematoxylin–Eosin Staining

Harvested mandibles (first molar region) were fixed in 10% buffered formalin, then were decalcified in 10% EDTA for 2 months. After dehydration, mandibles were embedded in paraffin and cut into sagittal section of 5 µm for HE staining, HE examination to see the number of osteoclasts and osteoblasts seen in five visual fields. Image acquisition was performed using a Nikon H600L microscope under ×200, ×400, and ×1,000 magnification, DS-Fi2 camera, and Nikon image system software.

### Immunohistochemical Evaluation

The demineralized and paraffin-embedded mandibles were cut into 5-μm sections and treated with 3% H _2_ O _2_ for 5 minutes at room temperature. Then, sections were blocked with ultra V block and incubated with prediluted RUNX2 monoclonal antibody (RUNX2 [27-K]: sc-101145) (Santa Cruz Biotechnology, Inc., United States), Osterix monoclonal antibody (OSX [F-3]: sc-393325) (Santa Cruz Biotechnology, Inc.), VEGF monoclonal antibody (VEGF [C-1]: sc-7269) (Santa Cruz Biotechnology, Inc.), mouse monoclonal anti-TRAP antibody (1:100, ABIN6654430, antibodies-online GmbH), mouse monoclonal anti-RANKL antibody (1:100, ABIN5611143, antibodies-online GmbH), and mouse polyclonal anti-OPG antibody (1:100, ABIN925985, antibodies-online GmbH) for 25 to 30 minutes at room temperature. All slides were incubated for 15 minutes at room temperature with secondary antibodies and counterstained with hematoxylin. The expression of RUNX2, Osterix, VEGF, TRAP, RANKL, and OPG were evaluated using the Remelle scale index (immunoreactive score [IRS]) which was the result of multiplying the percentage score of immunoreactive cells with the color intensity score of immunoreactive cells. The data for each sample are the IRS mean value in five fields of view on a ×200, ×400, and ×1,000 magnification. All examinations used a Nikon H600L light microscope equipped with a 300-megapixel DS-Fi2 digital camera and image processing software Nikon Image System ([negative = 0, low intensity = 1, moderate intensity = 2, and strong intensity = 3] × [negative = 0, positive cell < 10% = 1, positive cell 11–50% = 2, positive cell <51–80% = 3, positive cell > 80% = 4]).

### Statistical Analysis

All data are presented as the mean ± standard deviation. Statistical analysis was performed using the statistics package SPSS 22.0 (IBM, Armonk, New York, United States). Comparison between the groups was determined using one-way analysis of variance with least significant difference multiple comparison test and Kruskal–Wallis' test with Mann–Whitney's test. A *p* < 0.05 was considered to be statistically significant.

## Results

### Osteoporosis and DM Reduced RUNX2 Expression

**Fig. 1:**
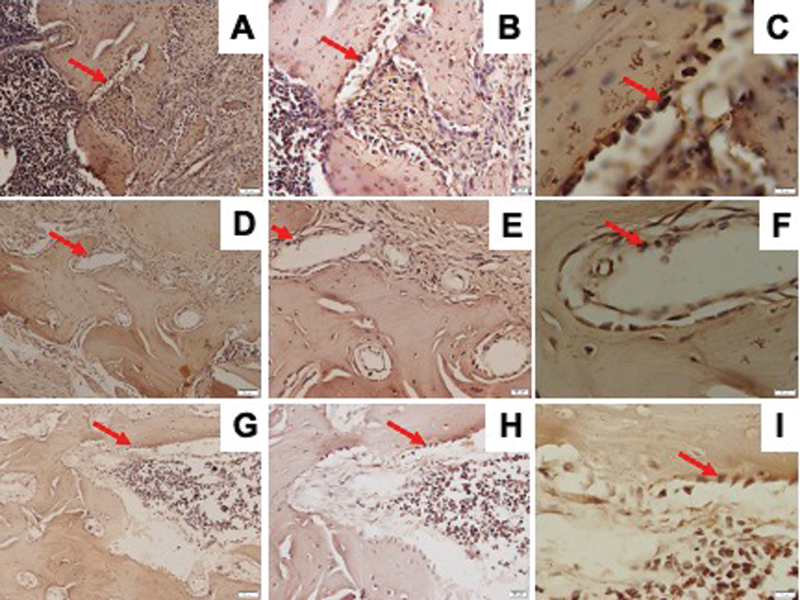
Representative images of immunohistochemical RUNX2 expression in the mandible of Wistar rats in diabetic group with ×200 magnification ( **A** ), ×400 magnification ( **B** ), ×1,000 magnification ( **C** ); osteoporotic group with ×200 magnification ( **D** ), ×400 magnification ( **E** ), ×1,000 magnification ( **F** ); and control group with ×200 magnification ( **G** ), ×400 magnification ( **H** ), ×1,000 magnification ( **I** ); and RUNX2-positive cells were observed (red arrow). RUNX2, Runt-related transcription factor 2.

**Fig. 2:**
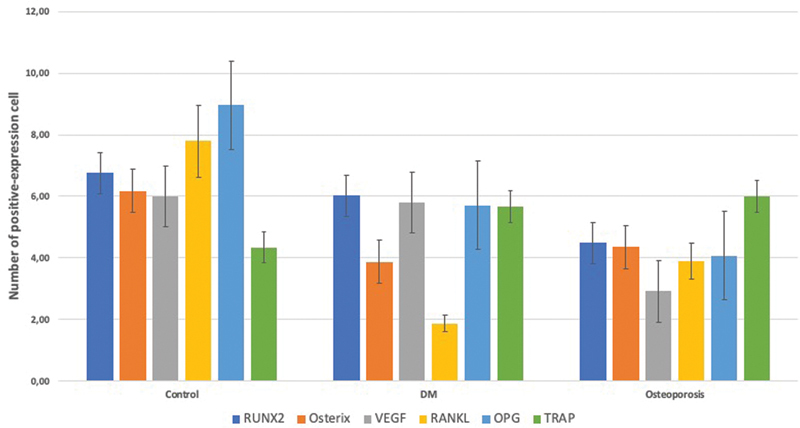
Quantitative analysis of immunohistochemical staining cell expressions. Data are presented as mean ± standard deviation. *n* = 9. DM, diabetes mellitus; OPG, osteoprotegerin; RUNX2, Runt-related transcription factor 2; RANKL, receptor activator of nuclear factor κB ligand; TRAP, tartrate-resistant acid phosphatase; VEGF, vascular endothelial growth factor.

RUNX2 expression observed by IHC staining is shown in Fig. 1 . The osteoporosis group showed significantly lower RUNX2 expression than the control and DM groups. The DM group showed a lower, but not statistically significant, expression of RUNX2 than the control group ( Fig. 2 ).

### Osteoporosis and DM Reduced Osterix Expression

**Fig. 3:**
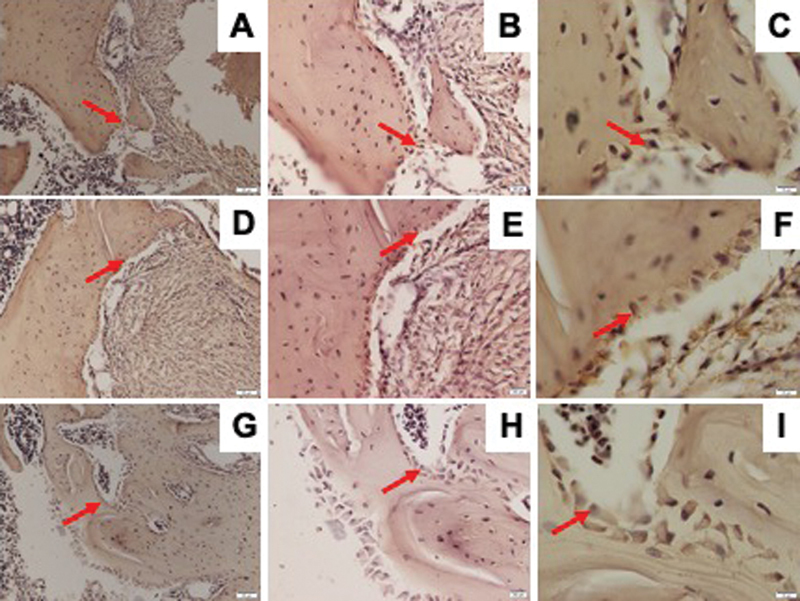
Representative images of immunohistochemical Osterix expression in the mandible of Wistar rats in diabetic group with ×200 magnification ( **A** ), ×400 magnification ( **B** ), ×1,000 magnification ( **C** ); osteoporotic group with ×200 magnification ( **D** ), ×400 magnification ( **E** ), ×1,000 magnification ( **F** ); and control group with ×200 magnification ( **G** ), ×400 magnification ( **H** ), ×1,000 magnification ( **I** ); and Osterix-positive cells were observed (red arrow).

Osterix expression was determined by IHC staining as shown in Fig. 3 . The osteoporosis and DM groups showed significantly lower Osterix expression than the control group. The DM group showed lower, but not statistically significant, expression of Osterix than the osteoporosis group ( Fig. 2 ).

### Osteoporosis and DM Reduced VEGF Expression

**Fig. 4:**
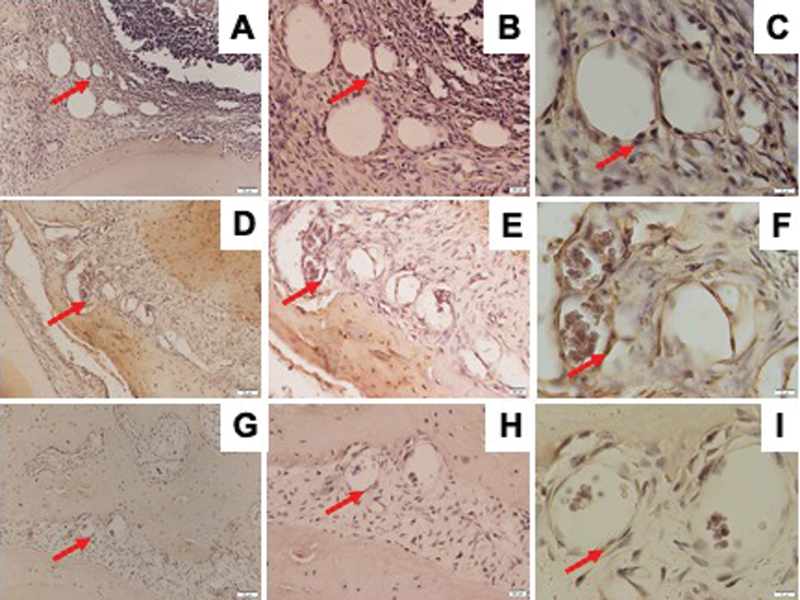
Representative images of immunohistochemical VEGF expression in the mandible of Wistar rats in diabetic group with ×200 magnification ( **A** ), ×400 magnification ( **B** ), ×1,000 magnification ( **C** ); osteoporotic group with ×200 magnification ( **D** ), ×400 magnification ( **E** ), ×1,000 magnification ( **F** ); and control group with ×200 magnification ( **G** ), ×400 magnification ( **H** ), ×1,000 magnification ( **I** ); and VEGF-positive cells were observed (red arrow). VEGF, vascular endothelial growth factor.

VEGF expression was determined by IHC staining as shown in Fig. 4 . The osteoporosis group showed significantly lower VEGF expression than the control and DM groups. The DM group showed lower, but not statistically significant, VEGF expression than the control group ( Fig. 2 ).

### Osteoporosis, but not DM, Promoted the Expression of RANKL/OPG Ratio

**Fig. 5:**
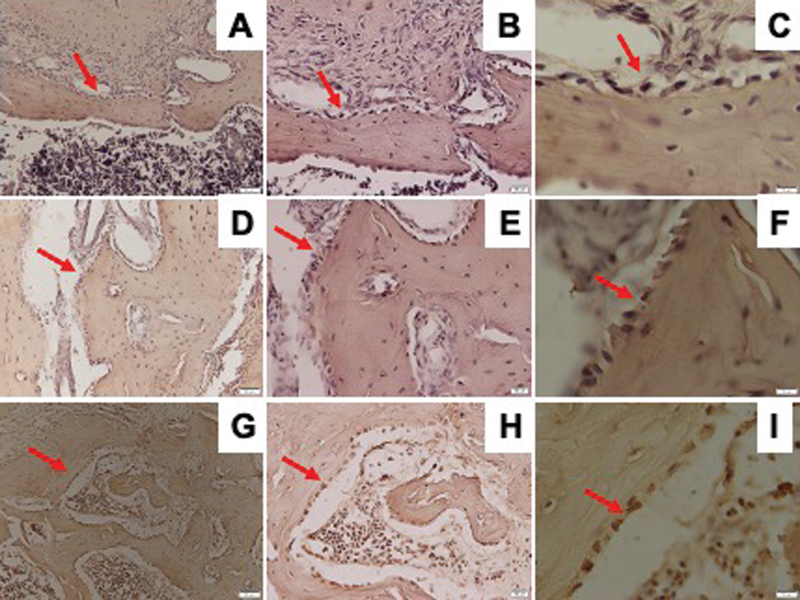
Representative images of immunohistochemical RANKL expression in the mandible of Wistar rats in diabetic group with ×200 magnification ( **A** ), ×400 magnification ( **B** ), ×1,000 magnification ( **C** ); osteoporotic group with ×200 magnification ( **D** ), ×400 magnification ( **E** ), ×1,000 magnification ( **F** ); and control group with ×200 magnification ( **G** ), ×400 magnification ( **H** ), ×1,000 magnification ( **I** ); and RANKL-positive cells were observed (red arrow). RANKL, receptor activator of nuclear factor κB ligand.

**Fig. 6:**
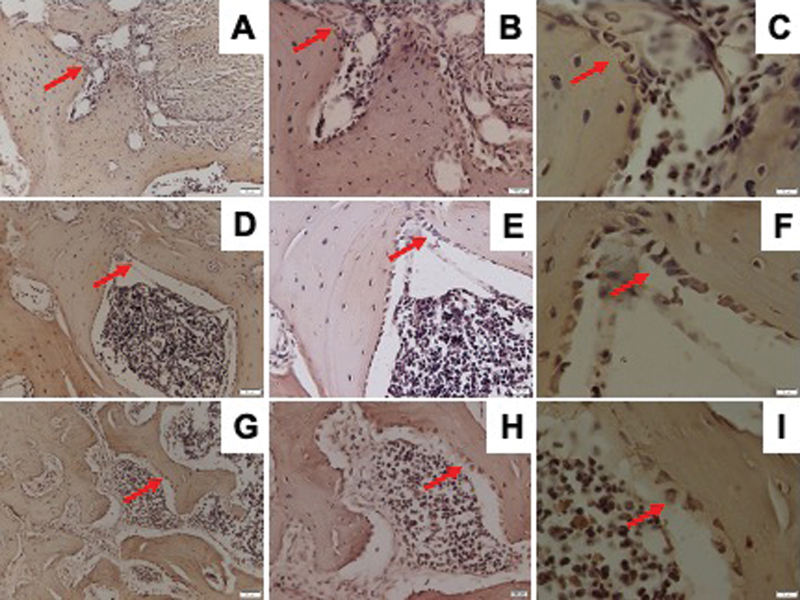
Representative images of immunohistochemical OPG expression in the mandible of Wistar rats in diabetic group with ×200 magnification ( **A** ), ×400 magnification ( **B** ), ×1,000 magnification ( **C** ); osteoporotic group with ×200 magnification ( **D** ), ×400 magnification ( **E** ), ×1,000 magnification ( **F** ); and control group with ×200 magnification ( **G** ), ×400 magnification ( **H** ), ×1,000 magnification ( **I** ); and OPG-positive cells were observed (red arrow). OPG, osteoprotegerin.

RANKL/OPG ratio has a pivotal role in osteoclast formation and activation. The numbers of RANKL and OPG-positive osteoblast were determined with IHC staining as shown in Figs. 5 and 6 . Compared with control group, either osteoporosis or DM led to decrease the number of OPG-positive cells in the mandible. However, the number of RANKL-positive cells was also downregulated in both osteoporotic and diabetic groups as compared with control group. Therefore, only in osteoporotic group, but not in diabetic group, the expression of RANKL/OPG ratio was increased ( Fig. 2 ).

### Both Osteoporosis and DM Did Not Have Any Effect in the Expression of TRAP

**Fig. 7:**
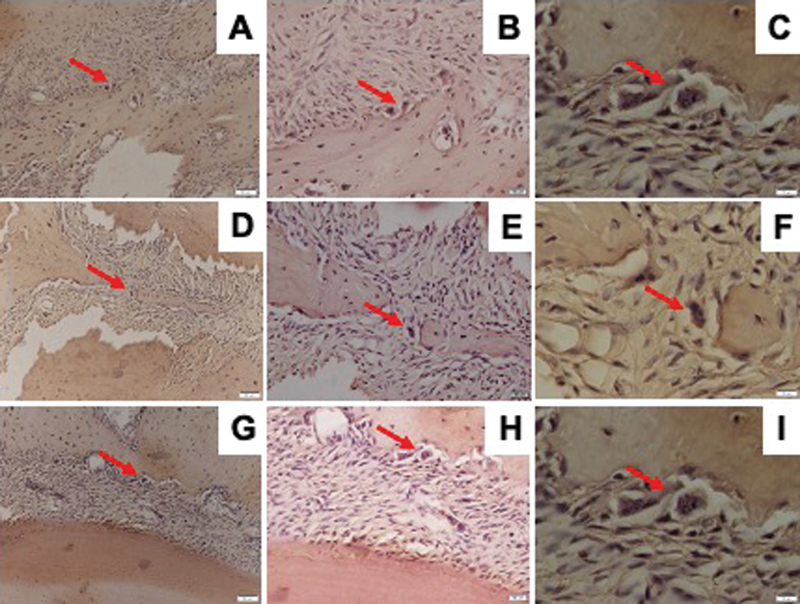
Representative images of immunohistochemical TRAP expression in the mandible of Wistar rats in diabetic group with ×200 magnification ( **A** ), ×400 magnification ( **B** ), ×1,000 magnification ( **C** ); osteoporotic group with ×200 magnification ( **D** ), ×400 magnification ( **E** ), ×1,000 magnification ( **F** ); and control group with ×200 magnification ( **G** ), ×400 magnification ( **H** ), ×1,000 magnification ( **I** ); and TRAP-positive cells were observed (red arrow). TRAP, tartrate-resistant acid phosphatase.

TRAP is abundantly expressed in osteoclasts and considered as a marker of osteoclast differentiation. 12 Therefore, we also observed TRAP-positive osteoclast using IHC staining as stated in Fig. 7 . Contrary to our expectations, neither osteoporosis nor DM upregulated the number of TRAP-positive osteoclasts ( Fig. 2 ).

### Osteoporosis and DM Reduced Osteoblast/Osteoclast Ratio

**Fig. 8:**
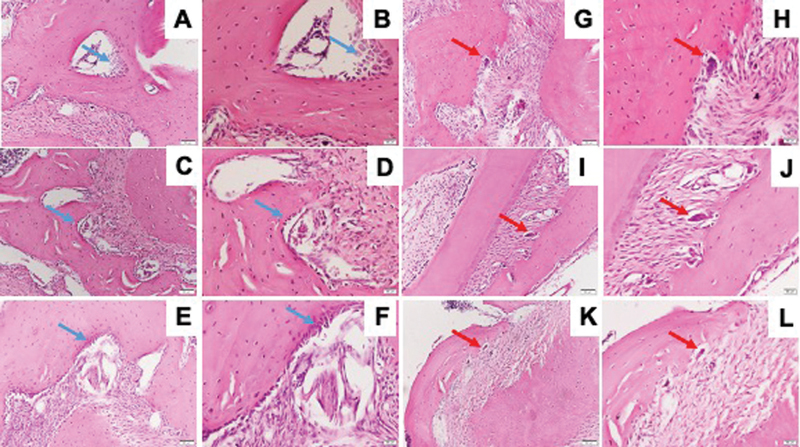
Representative images of HE stained. HE stained of osteoblast (blue arrow) and osteoclast (red arrow) in the mandible of Wistar rats in diabetic group with ×200 magnification ( **A, G** ), ×400 magnification ( **B, H** ); osteoporotic group with ×200 magnification ( **C, I** ), ×400 magnification ( **D, J** ); and control group with ×200 magnification ( **E, K** ), ×400 magnification ( **F, L** ). The arrow showed osteoblast at the bone surface. HE, hematoxylin–eosin.

**Fig. 9:**
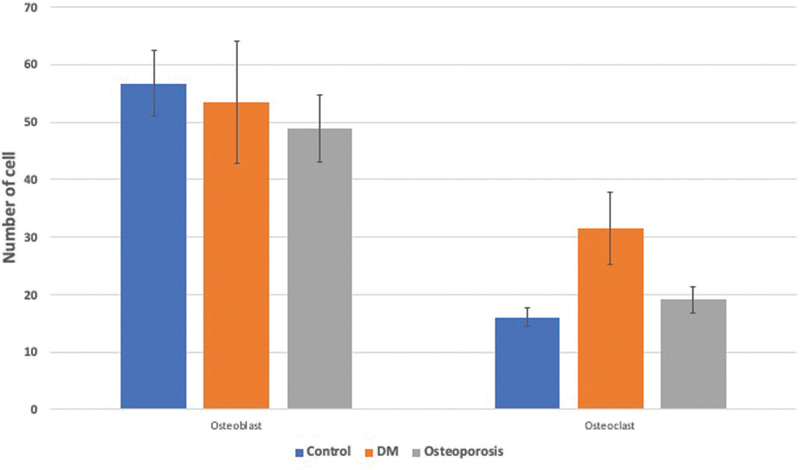
Quantitative analysis of histological staining for osteoblasts and osteoclasts. Data are presented as mean ± standard deviation. *n* = 9. DM, diabetes mellitus.

As shown in Fig. 8 , both osteoporosis and DM decreased the osteoblast/osteoclast ratio. The number of osteoblasts did not show any significant difference between osteoporosis, DM, and control groups. On the contrary, the number of osteoclasts was markedly increased in the mandible of diabetic group ( Fig. 9 ).

## Discussion

Hyperglycemic conditions initiate the formation of advanced glycation end-product (AGE) that can induce osteoblast apoptosis through the mitogen-activated protein kinase pathway. Receptor of AGE expression is also increased in osteoblasts in DM, thereby increasing the sensitivity of cells to the effects of AGEs. In addition, AGE was able to inhibit the differentiation of mesenchymal stem cells (MSCs) which are osteoblast precursors. 23 In addition, hyperglycemia can inhibit the Wnt/β-catenin signaling pathway where it also plays an important role in RUNX2-stimulated osteogenesis. 24

The reduction of RUNX2 could be due to the decrease in estrogen levels affecting the suppression of MSC production and the number of preosteoblasts so that the number of osteoblasts also decreases. The process of osteogenesis in osteoporosis has entered the final phase of mature bone formation so that the amount of RUNX2 expression has decreased. Under conditions of hyperglycemia, AGE was able to inhibit the differentiation of MSCs into osteoblasts. It is possible that the effect of estrogen on the suppression of MSC production is stronger than that of AGE which inhibits MSC differentiation. However, this finding needs to be performed in more in-depth research to be able to confirm this. 2

In a previous study conducted by Hendrijantini et al 2 (2019), low levels of estrogen in the osteoporosis group affected the decrease in the number of osteoblasts. One of the number of osteoblasts depends on transforming growth factor (TGF)-β1. The role of TGF-β1 in the process of bone formation inhibits the expression of RUNX2 thereby preventing maturation of osteoblasts from differentiating into osteocysts. 2

The diabetes group had lower Osterix expression than the control group. This is in accordance with research conducted by Rios-Arce et al (2020) showing that the diabetes group had lower Osterix expression than the control group. High blood glucose in the diabetic group decreased extracellular signal-regulated kinase (ERK) phosphorylation. The ERK pathway is known as a key stimulator of osteoblast differentiation. 25

Osteoporosis model group was ovariectomized which causes estrogen levels in the body to decrease so that the proliferation and differentiation of human bone marrow–derived MSCs also decreases through the Notch signaling pathway. Notch signaling plays a role in the proliferation and early differentiation of osteoblasts. 26 The condition of DM can cause a decrease in MSC differentiation. Postmenopausal estrogen deficiency may also lead to decreased MSC differentiation. This is the reason why there may be no difference in Osterix expression values in the DM and osteoporosis groups. 27 28 29

VEGF is a specific endothelial growth factor that stimulates endothelial cell proliferation and differentiation, increases vascular permeability, mediates endothelial vasodilation, and participates in interstitial matrix remodeling.

The decrease in the hormone estrogen caused by ovariectomy or menopause can increase body fat mass. Visceral fat area is significantly increased in postmenopausal women compared with premenopausal women. It is known that glucocorticoids have an important role in the distribution of body fat mass. Glucocorticoids inhibit the production of cyclooxygenase-2 and prostaglandin E2 (PGE2). So that PGE2 which has the ability to synthesize VEGF is inhibited. 30

Accordance with research conducted by Zhang et al (2018), chronic hyperglycemia can stimulate the synthesis and secretion of VEGF. 31 VEGF accumulation can lead to microvascular complications. The main stimulus for VEGF production is cellular hypoxia. Hyperglycemia acts as a toxin to the endothelium by increasing oxidative stress. The high concentration of glucose in the blood increases the production of vasoconstrictors, especially endothelin-1. In osteoporosis, there is a decrease in the hormone estrogen which causes high levels of glucocorticoids in the body. Glucocorticoids can inhibit the production of PGE2, where PGE2 plays a role in VEGF synthesis. These findings can explain why the DM expression value is higher than the osteoporosis group. 30 31

The process of bone remodeling is also related to the RANKL/OPG ratio. The interaction between RANK and RANKL signifies the initiation of osteoclastogenesis and activation of osteoclasts. OPG is known as an osteoclast inhibiting factor. OPG is a binding receptor and a competitor for RANK receptors to bind to RANKL. OPG binding to RANKL inhibits osteoclastogenesis, thereby inhibiting bone resorption. The OPG–RANKL bond offsets the effect of the RANK–RANKL bond. Bone formation can be associated with increased OPG expression or reduced RANKL expression as indicated by a decrease in the RANKL/OPG ratio. Conversely, a decrease in OPG expression or an increase in RANKL expression can lead to pathological bone resorption characterized by an increase in the RANKL/OPG ratio. 16 18 32

This study found that the value of the RANKL/OPG ratio in the DM group was significantly lower than in the osteoporosis and control groups. This is probably caused by hyperglycemia which can cause a significant decrease in the RANKL/OPG ratio produced by osteoblasts. Sassi et al found that there was a decrease in RANKL levels in DM patients. Hu et al also found that high glucose levels inhibited RANKL-induced osteoclastogenesis which significantly reduced RANKL gene expression during bone regeneration. 33 34 35

This study found that the TRAP expression in the three study groups did not show a significant difference. Several previous studies on DM also showed varied results, Lu et al found that mice treated with DM showed a higher TRAP expression value than the control group. In contrast, Hu et al found that mature osteoclast biomarkers, including TRAP, were lower in DM group mice. Different results were also shown by Xu et al who found that TRAP serum values in DM mice did not have a significant difference with non-DM mice. The diversity of the results of this study is likely due to various factors that can affect the variability of the study, such as differences in research design, methodology, and other unknown factors. 33 36 37 38

Several previous studies on osteoporosis have also shown varied results. Halleen et al found that serum TRAP in postmenopausal women was higher than in premenopausal women. In contrast, Verit et al found no significant changes in serum TRAP in both postmenopausal women with osteoporosis, postmenopausal women without osteoporosis, and premenopausal women. Kim et al also did not find any significant changes in the expression of TRAP in the ovariectomy group mice with the control mice. This is probably due to the duration of osteoporosis after the ovariectomy. Miyazaki et al found that differences in expression of TRAP activity were more pronounced at study duration of less than 8 weeks. In this study, specimen collection from experimental animals was performed at week 12, and this factor is probably the reason that TRAP expression in the osteoporosis group did not differ significantly from that of the control. 39 40 41 42

The limitation of this study was the difference of observation time between osteoporosis and DM condition; therefore, the development of those diseases can be different. The future study was expected to have same and longer observation time so that the pathway and mechanism of DM and osteoporosis in mandible can be more understandable and prevented.

## Conclusion

The results of this study indicate that DM and osteoporosis can induce changes in the bone remodeling pathway which are represented by a decrease in osteoblast biomarkers and an increase in osteoclast biomarkers.
